# Ketamine exacerbates cortical neuroapoptosis under hyperoxic conditions by upregulating expression of the N-methyl-D-aspartate receptor subunit NR1 in the developing rat brain

**DOI:** 10.1186/s12871-018-0511-y

**Published:** 2018-05-10

**Authors:** Changyi Wu, Jun Wang, Xiangyang Guo, Ying Zhang

**Affiliations:** 1 Department of Anesthesiology, Peking University Third Hospital, Beijing, 100191 People’s Republic of China; 2 Department of Rehabilitation, China Rehabilitation Research Center, Boai Hospital, School of Rehabilitation Medicine, Capital Medical University, 10 North Road, Fengtai District, Beijing, 100077 People’s Republic of China

**Keywords:** NMDA receptor antagonist, Ketamine, Hyperoxia, Apoptosis, Newborn, Rat

## Abstract

**Background:**

Ketamine and hyperoxia are widely used in obstetric and pediatric settings. Either ketamine or hyperoxia has been reported to cause neuroapoptosis in the developing brain, and ketamine-induced neuronal apoptosis may involve a compensatory upregulation of the N-methyl-D-aspartate (NMDA) receptor NR1 subunit. This study investigated the impact of ketamine administration under hyperoxic conditions on cortical neuroapoptosis and NR1 subunit expression in the infant rat brain.

**Methods:**

Male, 7-day-old rats were randomly allocated to four groups: control, ketamine, hyperoxia, and ketamine + hyperoxia (*n* = 18 per group). Rats in the control and ketamine groups received subcutaneous injections of either vehicle (saline) or ketamine (50 mg/kg) in room air (21% oxygen). The hyperoxia and ketamine + hyperoxia groups were exposed to 60% oxygen for 2 h after receiving saline or ketamine. Physiological parameters and arterial oxygen saturation were observed. Neuronal apoptosis and the expressions of NR1 mRNA and protein in the frontal cortex were also examined by transferase dUTP nick end labeling (TUNEL) assays, qPCR and Western blot, respectively.

**Results:**

Ketamine alone had no effect on paO_2_ (*P* > 0.05)_,_ but pups exposed to hyperoxia or hyperoxia + ketamine had significantly greater paO_2_ values compared to control animals (*P* < 0.01). Animals exposed to ketamine and ketamine + hyperoxia showed higher apoptotic scores, mRNA and protein expression levels of NR1 than control animals (*P* < 0.01), and ketamine + hyperoxia caused a significantly greater increase than ketamine alone (*P* < 0.01).

**Conclusions:**

These data suggest that ketamine administration under hyperoxic conditions exacerbates cortical neuroapoptosis in the developing brain, which may be closely associated with an enhancement in NMDA receptor NR1 subunit expression.

## Background

Ketamine, an N-methyl-D-aspartate (NMDA) receptor antagonist, is widely used as an anesthetic, analgesic, and sedative in obstetric and pediatric settings. However, it has been suggested to induce neuroapoptosis in the developing brain [1]. More recent preclinical studies also indicate that ketamine use in newborn rats and rhesus monkeys leads to widespread neurodegeneration and long lasting cognitive deficits [2, 3]. Developmental neurotoxicity is therefore regarded as a complication of ketamine relevant to its use in pediatrics.

Supraphysiological oxygen concentrations (hyperoxia) are widely used in neonatal medicine for resuscitation, pulmonary hypertension, and respiratory distress syndrome. There is clear evidence that hyperoxia is toxic to developing lungs and retinas [4, 5], and it can also induce massive apoptotic neurodegeneration in the developing brain [6–8]. Although hyperoxia is commonly used to prevent and treat hypoxemia during ketamine anesthesia in pediatric surgery [9], there is little information about the potential effects of this combination on developmental neurotoxicity.

The NMDA glutamate receptor is a ligand-gated ion channel consisting of an essential NR1 subunit, one or more regulatory NR2 subunits (NR2A–D), and an NR3 subunit [10]. The NR1 subunit is widely distributed throughout the brain and plays an essential role in maintaining NMDA channel function. NMDA receptor blockade and developmental neurotoxicity have been investigated extensively. Ketamine administration to rat forebrain cultures induces apoptotic cell death by upregulating NR1 subunit expression [11]. Although little is known about the pathophysiological significance of the NMDA receptor in hyperoxia-induced apoptotic neurodegeneration in the developing brain, it has been reported that hyperoxia exposure can increase NR2D subunit expression in neonatal rat lung [12].

The aim of the present study was to evaluate the influence of ketamine combined with hyperoxia on cortical neuroapoptosis and the expression of the NR1 NMDA receptor subunit in the developing rat brain.

## Methods

### Animal experiments

Animal experiments were performed according to the guidelines of the Peking University Health Center Ethics Committee on Animal Care. Seven-day-old male Sprague Dawley rat pups (average body weight 12–16 g) were obtained from the Animal Center of Peking University Health Center, Beijing, China, and randomly allocated to four groups: control, ketamine, hyperoxia, and ketamine + hyperoxia (*n* = 18 per group). Rats in the control and ketamine groups received subcutaneous injections (10 ml/kg) of either vehicle (saline) or ketamine (5 mg/ml, 50 mg/kg; Hengrui Medicine Co., Jiangsu, China) in room air (21% oxygen). The hyperoxia and ketamine + hyperoxia groups were exposed to 60% oxygen for 2 h in a Plexiglas chamber immediately after receiving subcutaneous injections of either saline or ketamine (50 mg/kg). The oxygen concentration in the chamber was monitored continuously. Humidity was maintained at > 80% and CO_2_ was removed by soda lime absorption. The treatment was performed in a temperature-controlled container at 36.7 °C and body temperature was monitored with a MicroTherma 2 T device and a neonatal rat rectal probe (Braintree Scientific, MA, USA). After the treatment, the pups were placed back with their dams in room air at 22 ± 1 °C.

### Blood gas measurements

Blood gas levels were obtained from each group (*n* = 6 per group) 2 h after the subcutaneous injections. Animals exposed to hyperoxia were decapitated in the oxygen chamber after hyperoxia exposure. The blood sample was obtained by transcardiac puncture from the left ventricle with a heparinized 32-gauge hypodermic needle. Bicarbonate concentration (mM), oxygen saturation (SaO_2,_ %), pH, partial pressure of carbon dioxide (paCO_2_, mmHg) and partial pressure of oxygen (paO_2_, mmHg) were measured with a blood analyzer (Cobas b123; Roche, Rotkreuz, Switzerland) immediately after blood collection.

### Tissue sampling

Animals were sacrificed with intraperitoneal sodium pentobarbital (100 mg/kg) 24 h after the subcutaneous injection. The frontal cortices from each group (*n* = 6 per group) were rapidly isolated and frozen in liquid nitrogen before being processed for quantitative reverse transcription polymerase chain reaction (qPCR) and Western blotting. The remaining rat pups (*n* = 6 per group) were transcardially perfused with 0.9% saline and 4% paraformaldehyde in 0.1 M phosphate buffer (pH 7.2) for the detection of neuronal apoptosis by terminal deoxynucleotide transferase-mediated dUTP nick end-labeling (TUNEL).

### In situ apoptosis analysis

The extent of apoptosis in the paraffin-embedded coronal brain sections of animals from all the groups was analyzed using the DeadEnd Fluorometric TUNEL assay (Promega, Madison, WI, USA) according to the manufacturer’s protocol. In brief, the paraffin-embedded tissue sections were de-paraffinized, rehydrated, treated with proteinase K working solution, and permeabilized. Permeabilized tissue sections were incubated with the TUNEL reaction mixture in a humidified atmosphere for 60 min at 37 °C in the dark. Sections were counterstained for nuclei with DAPI (Invitrogen, Carlsbad, CA, USA) and observed under a confocal laser-scanning microscope (Olympus FV1000). Five sections from each brain were taken and five microscopic fields per section were analyzed. The apoptotic index was calculated as percentage of DAPI-stained, TUNEL-positive cells to the total number of cells.

### qPCR

Total RNA was extracted using TRIzol reagent (Invitrogen, Darmstadt, Germany) and first-strand cDNA synthesis was performed using random primers and SuperScript III Reverse Transcriptase (Invitrogen, Darmstadt, Germany); 1 μg total RNA was converted into cDNA. qPCR for the NR1 gene and the housekeeping gene β-actin was performed using TaqMan probes and the CFX96 Real-Time PCR system (Bio-Rad, Hercules, CA). The following primers were used: NR1 (NM_017010; forward, 5′-CTT CCT CCA GCC ACT ACC C-3′; reverse, 5′-AGA AAG CAC CCC TGA AGC AC-3′); β-actin (NM_031144; forward, 5′-TGA CAG GAT GCA GAA GGA GA-3′; reverse, 5′-TAG AGC CAC CAA TCC ACA CA-3′). The thermal cycle conditions were 10 min at 95 °C, 2-step PCR for 40 cycles of 95 °C for 15 s, and a final incubation at 60 °C for 1 min. The threshold cycle (Ct) value for each well and PCR efficiency (E) were determined for each gene. The formula 2^−ΔΔCt^ was used to normalize gene expression to the control group. Samples from each animal were tested three times to reduce variability.

### Western blot

Protein samples from frontal cortices were prepared using a protein extraction kit (Nanjing Jiancheng Biochemistry Co., Nanjing, China) containing 20 mM Tris, 150 mM NaCl, 1 mM EDTA, 1 mM EGTA, 1% Triton X-100, 2.5 mM sodium pyrophosphate, 1 mM Na_3_VO_4_, 1 mM β-glycerophosphate, 1 μg/ml leupeptin and aprotinin. Protein concentrations were determined using a BCA protein assay kit (Nanjing Jiancheng Biochemistry Co.). Protein extracts (25 μg) were fractionated on 12% sodium dodecyl sulfate polyacrylamide gel and then transferred to a nitrocellulose membrane. Two hours after blocking with PBS (pH 7.4) plus 0.25% Tween 20 (*v*/v) and 5% non-fat dried milk, the membrane was probed with an anti-NR1 monoclonal antibody (1:300, BD Biosciences Pharmingen, California, USA) overnight at 4 °C. Next, the membrane was incubated with horseradish peroxidase-conjugated anti-mouse secondary antibody (1:2000; Santa Cruz Biotechnology Inc., Santa Cruz, CA, USA) for 1 h at room temperature. Antibody binding was visualized with a chemiluminescence system and short exposure of the membrane to X-ray films (Bio-Rad, California, USA). The ratios of densitometric value of NR1 to β-actin represented the NR1 expression level.

### Statistical analysis

All values are expressed as the mean ± standard deviation (SD). Differences were analyzed by one-way analysis of variance (ANOVA) with the Least Significant Difference or Tamhane′s T2 multiple comparison post hoc tests using SPSS 15.0 statistical software (SPSS Inc., Chicago, IL, USA). Statistical significance was defined at *P* < 0.05.

## Results

### Physiological parameters

Animals that received ketamine or ketamine + hyperoxia did not lose consciousness, but performed paddling movements of their paws for approximately 40–60 min. Feeding habits, body weight, body temperature, and breathing patterns did not differ among the four groups.

### Arterial oxygen saturation

Ketamine alone had no effect on paO_2_ compared with the control group (*P* > 0.05). Animals exposed to hyperoxia or hyperoxia + ketamine had significantly greater paO_2_ values compared to control animals (*P* < 0.01), but there was no significant difference between the two hyperoxia groups (*P* > 0.05). No changes in bicarbonate concentration, SaO_2_, pH or paCO2 were observed among the four groups (Table 1).

**Table 1:** Blood gas profiles

Group	pH	paO_2_ (mmHg)	paCO_2_ (mmHg)	HCO_3_^−^ (mEq/L)	SaO_2_ (%)
Control (n = 6)	7.41 ± 0.07	89.8 ± 3.7	25.3 ± 3.1	17.5 ± 3.3	97.5 ± 1.1
Ketamine (n = 6)	7.40 ± 0.09	91.5 ± 3.8	26.3 ± 3.8	19.1 ± 3.3	98.0 ± 1.4
Hyperoxia (n = 6)	7.41 ± 0.08	242.3 ± 12.7^**^	24.2 ± 4.7	18.1 ± 3.9	98.2 ± 1.5
Ketamine+hyperoxia (n = 6)	7.43 ± 0.06	244.7 ± 9.1^**^	23.5 ± 3.4	19.2 ± 2.7	97.8 ± 1.3

Data are the mean ± SD, ^**^P < 0.01 vs. control

### Ketamine with and without hyperoxia promotes apoptosis in the frontal cortex

**Fig. 1:**
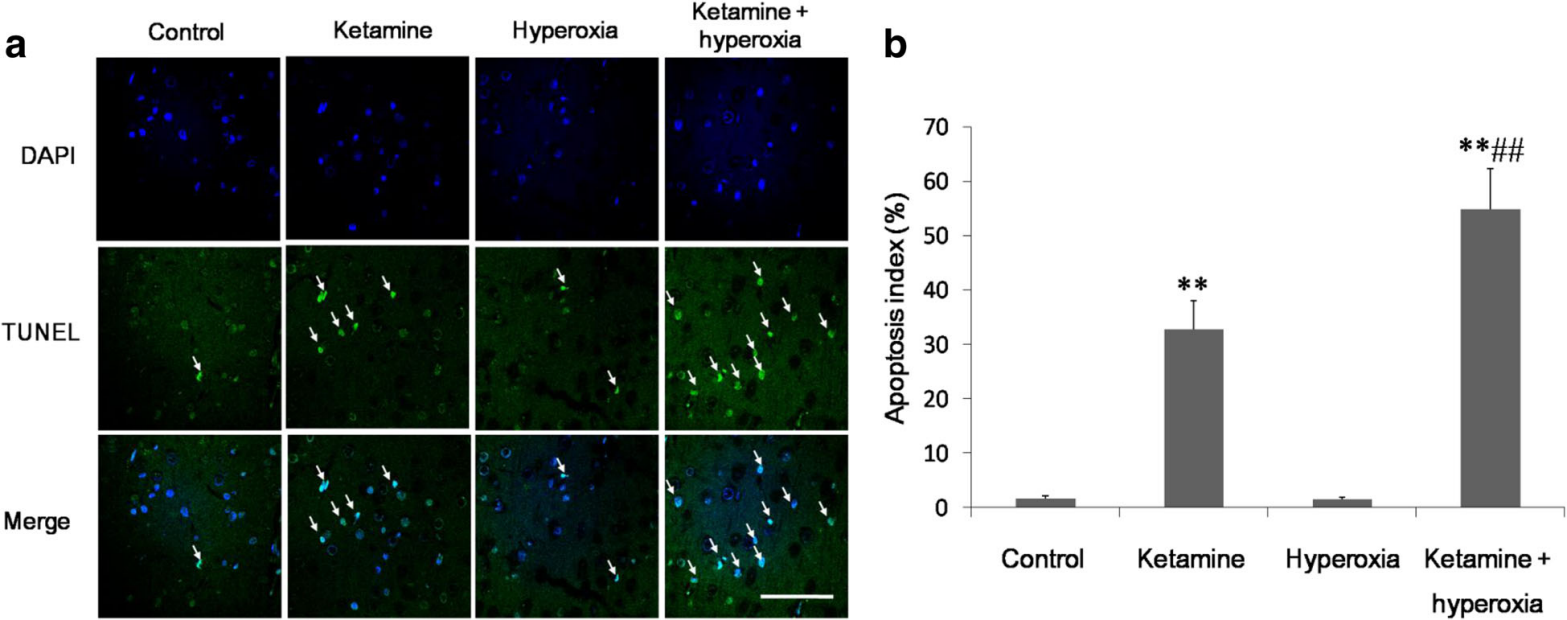
Ketamine or ketamine + hyperoxia promotes apoptosis in the frontal cortex. **a** TUNEL assay on paraffin-embedded coronal sections of frontal cortex from rat pups. Green fluorescence indicates TUNEL-positive cells. Blue fluorescence indicates DAPI staining of nuclei. Scale bar: 20 μm. **b** Quantification of TUNEL-positive cells in frontal cortex (mean ± SD; *n* = 6 rats per group). The apoptotic index was calculated as the number of DAPI- and TUNEL-positive cells divided by the number of DAPI-positive cells. **P < 0.01 vs. control; ^##^*P* < 0.01 vs. ketamine

Pups exposed to hyperoxia alone had apoptotic scores similar to those of littermates from the control group (*P* > 0.05). However, those that received ketamine or ketamine + hyperoxia had more apoptotic cell death than controls (*P* < 0.01). Furthermore, ketamine + hyperoxia induced more extensive apoptosis than ketamine alone (*P* < 0.01) (Fig. 1).

### Ketamine with and without hyperoxia upregulates NR1 mRNA and protein expression in the frontal cortex

**Fig. 2:**
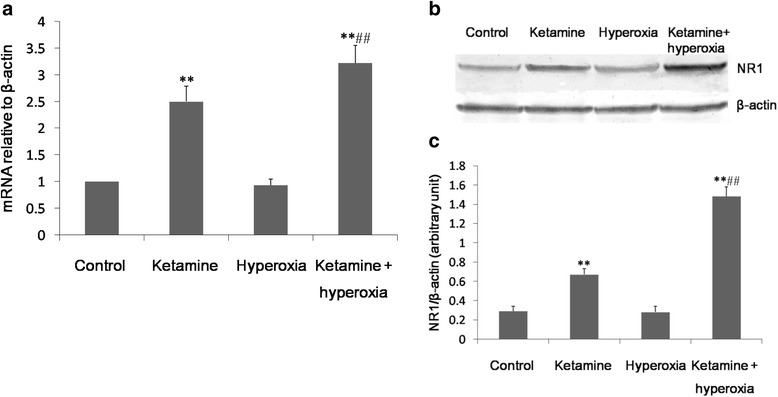
Ketamine or ketamine + hyperoxia upregulates NR1 subunit mRNA and protein expression in the frontal cortex. **a** qPCR analysis of NR1 mRNA expression in the frontal cortex. **b** Western blot analysis of NR1 subunit protein in the frontal cortex; β-actin was used as the internal standard. **c** Relative band densities of NR1 subunit after the different treatments. Values are expressed as mean ± SD (n = 6 per group). **P < 0.01 vs. control; ^##^*P* < 0.01 vs. ketamine

Compared with the control group, hyperoxia alone had no effect on the mRNA and protein expression of the NR1 subunit (*P* > 0.05). However, animals exposed to ketamine and ketamine + hyperoxia showed higher mRNA and protein expression of NR1 than control animals (*P* < 0.01). Furthermore, ketamine + hyperoxia caused a significantly greater increase in the expression of NR1 subunit mRNA and protein than ketamine alone (*P* < 0.01) (Fig. 2).

## Discussion

It is well known that the developing brain is highly sensitive to the apoptogenic action of general anesthetics at the peak of synaptogenesis. However, the brain growth spurt period (i.e. synaptogenesis) occurs at different times in different species. In the rat, the brain growth spurt is reported to start postnatally, peak at postnatal day 7, and end by 25 postnatal days [13]. In humans, the comparable period lasts from the third trimester to the second or third year after birth. The results of the present study reveal that ketamine exacerbates cortical neuroapoptosis by itself and under hyperoxic conditions by upregulating the expression of the NMDA glutamate receptor NR1 subunit in 7-day-old rat pups.

In 1999, it was first reported that ketamine caused neurodegeneration in the developing brain [1]. Further studies have also confirmed that high or repeated doses of ketamine can induce neuroapoptosis in many kinds of in vitro and in vivo models, including in mice, rats, and monkeys [14–17]. However, the developmental neurotoxicity of a single ketamine injection remains controversial. A study by Hayashi et al. found that a single injection of ketamine to 7-day-old rats at doses of 25–75 mg/kg did not trigger neuroapoptosis [18]. In contrast, we show here, using the TUNEL assay, that a single subcutaneous injection of ketamine (50 mg/kg) significantly induced apoptotic neurodegeneration in the frontal cortex of 7-day-old rats. This discrepancy is likely caused by the difference in methods. Hayashi et al. used the De Olmos silver staining method to detect neuronal degeneration 24 h after ketamine administration. This is an appropriate method for mapping patterns of neurodegeneration (argyrophylia) resulting from prolonged or very high doses of drug exposure, but is not reliable for detecting or quantifying subtle increases in neuroapoptosis. Similar to our results, Ullah et al. showed that only a single subcutaneous injection of ketamine (40 mg/kg) could cause extensive nueroapoptosis in the infant rat forebrain [19]. Of course, it is possible that the adverse effects of ketamine, such as hypoxia and ischemia, may have contributed to the neuroapoptosis we observed, but the arterial blood gas analysis results rule out this possibility.

In addition to anesthetics, oxygen, which is widely used during general anesthesia, constitutes another possible contributing neurotoxic factor. Interestingly, the vulnerability to oxygen neurotoxicity is confined to the first 2 weeks of life, coinciding with the brain growth spurt. Few studies have examined the effects of hyperoxia on neurodegeneration in the developing brain. One clinical study showed that hyperoxia increased the risk of cerebral palsy in preterm infants [20]. An animal study showed that a 2-h exposure to 80% oxygen caused significant cortical neuroapoptosis in 7-day-old rat pups at 24 h, but exposure to 60% oxygen over 12 h did not [6]. Consistent with those findings, the results of the present study demonstrate that an exposure to 60% oxygen for 2 h could not, on its own, trigger cortical neuroapoptosis in rat pups. Furthermore, our results show that hyperoxia + ketamine induced a significantly greater increase in neuroapoptosis than ketamine alone. Together, these data suggest that hyperoxia-induced neuroapoptosis is associated with oxygen concentration and the duration of exposure to hyperoxia, and that hyperoxia may potentiate ketamine-induced neuroapoptosis in the developing brain.

To date, the mechanisms underlying ketamine-induced neurotoxicity in the developing brain have not been completely elucidated. However, in vitro and in vivo studies suggest that ketamine-induced neuroapoptosis in the immature central nervous system may involve a compensatory upregulation of the NMDA receptor NR1 subunit and subsequent overstimulation of the glutamatergic system by endogenous glutamate [11, 21]. It has been shown that NMDA receptor NR1 subunit mRNA is prominently expressed in the frontal cortex [21]. So, the frontal cortex is the brain region most vulnerable to ketamine-induced neurotoxicity [15]. Consistent with those previous findings, the present study demonstrated that a single injection of ketamine significantly increased NR1 mRNA and protein expression in the frontal cortex of rat pups. Moreover, in contrast to the effect of hyperoxia in the lung [12], we did not observe enhanced NR1 subunit expression in the frontal cortex after hyperoxia exposure, indicating that this action of hyperoxia may be organ-specific. Interestingly, the results of the present study also showed that combined administration of ketamine and hyperoxia caused a significantly greater increase in NR1 expression than ketamine alone. These data suggest that an increase in NR1 subunit expression is associated with the elevated neurotoxicity of ketamine under hyperoxic conditions. Of course, it is necessary to note that the current data are inadequate to testify the direct relationship between the ketamine combined with hyperoxia and the increased expression of NR1 subunit. So, more studies are needed to illuminate the exact mechanism by which ketamine with or without hyperoxic conditions further upregulates NR1 expression.

## Conclusion

In summary, the present data demonstrate that ketamine administration exacerbates cortical neuroapoptosis under hyperoxic conditions in the developing brain, which may be closely associated with enhanced NMDA receptor NR1 subunit expression. These findings provide preliminary evidence demonstrating the safety of ketamine administration under hyperoxic conditions in the developing brain.

## Abbreviations

NMDA: N-methyl-D-aspartate
qPCR: Quantitative reverse transcription polymerase chain reaction
TUNEL: Transferase dUTP nick end labeling
